# Factors associated with the prescribing of high-dose opioids in primary care: a systematic review and meta-analysis

**DOI:** 10.1186/s12916-020-01528-7

**Published:** 2020-03-30

**Authors:** Georgia C. Richards, Kamal R. Mahtani, Tonny B. Muthee, Nicholas J. DeVito, Constantinos Koshiaris, Jeffrey K. Aronson, Ben Goldacre, Carl J. Heneghan

**Affiliations:** 1 Centre for Evidence-Based Medicine, Nuffield Department of Primary Care Health Sciences, University of Oxford, Radcliffe Observatory Quarter, Woodstock Road, Oxford, OX2 6GG UK; 2 Nuffield Department of Primary Care Health Sciences, University of Oxford, Radcliffe Observatory Quarter, Woodstock Road, Oxford, OX2 6GG UK; 3 EBMDatalab, Nuffield Department of Primary Care Health Sciences, University of Oxford, Radcliffe Observatory Quarter, Woodstock Road, Oxford, OX2 6GG UK

**Keywords:** Opioids, High dose, Primary care, Systematic review, Benzodiazepines, Depression, Emergency department

## Abstract

**Background:**

The risks of harms from opioids increase substantially at high doses, and high-dose prescribing has increased in primary care. However, little is known about what leads to high-dose prescribing, and studies exploring this have not been synthesized. We, therefore, systematically synthesized factors associated with the prescribing of high-dose opioids in primary care.

**Methods:**

We conducted a systematic review of observational studies in high-income countries that used patient-level primary care data and explored any factor(s) in people for whom opioids were prescribed, stratified by oral morphine equivalents (OME). We defined high doses as ≥ 90 OME mg/day. We searched MEDLINE, Embase, Web of Science, reference lists, forward citations, and conference proceedings from database inception to 5 April 2019. Two investigators independently screened studies, extracted data, and appraised the quality of included studies using the Quality Assessment Tool for Observational Cohort and Cross-Sectional Studies. We pooled data on factors using random effects meta-analyses and reported relative risks (RR) or mean differences with 95% confidence intervals (CI) where appropriate. We also performed a number needed to harm (NNT_H_) calculation on factors when applicable.

**Results:**

We included six studies with a total of 4,248,119 participants taking opioids, of whom 3.64% (*n* = 154,749) were taking high doses. The majority of included studies (*n* = 4) were conducted in the USA, one in Australia and one in the UK. The largest study (*n* = 4,046,275) was from the USA. Included studies were graded as having fair to good quality evidence. The co-prescription of benzodiazepines (RR 3.27, 95% CI 1.32 to 8.13, *I*^2^ = 99.9%), depression (RR 1.38, 95% CI 1.27 to 1.51, *I*^2^ = 0%), emergency department visits (RR 1.53, 95% CI 1.46 to 1.61, *I*^2^ = 0%, NNT_H_ 15, 95% CI 12 to 20), unemployment (RR 1.44, 95% CI 1.27 to 1.63, *I*^2^ = 0%), and male gender (RR 1.21, 95% CI 1.14 to 1.28, *I*^2^ = 78.6%) were significantly associated with the prescribing of high-dose opioids in primary care.

**Conclusions:**

High doses of opioids are associated with greater risks of harms. Associated factors such as the co-prescription of benzodiazepines and depression identify priority areas that should be considered when selecting, identifying, and managing people taking high-dose opioids in primary care. Coordinated strategies and services that promote the safe prescribing of opioids are needed.

**Study registration:**

PROSPERO, CRD42018088057

## Background

The increase in opioid prescribing for long-term pain conditions led to more people taking opioids at higher doses. High doses of opioids are associated with greater morbidity [1, 2], mortality [3], and cost [4]. Despite this, the prescribing of high-dose opioids remains relatively common in high-income countries [5–7].

High doses of opioids are indicated in palliative care and cancer pain. However, there is little evidence on the effectiveness and safety of opioids at high doses for people with chronic pain. A Cochrane overview of systematic reviews on high-dose opioids for chronic non-cancer pain found no studies or data that could be extracted [8]. Clinical guidelines thus caution against prescribing high doses and recommend reducing or withdrawing opioids when the risk of harm outweighs the chance of benefit [9–11]. However, these recommendations have come under scrutiny [12]. The adoption of strict guidelines may reduce access to primary care which could lead to unintended consequences such as the conversion to illicit opiates and reduce the management of comorbidities such as depression [13, 14].

Most people with chronic pain are managed in primary care settings [15]. However, most primary care physicians perceive chronic non-cancer pain to be the most challenging condition to treat [16, 17]. While primary care remains the ideal setting to identify and manage such patients, there is little evidence on best practices for managing people taking high doses of opioids in primary care [18]. Thus, understanding who is taking high doses and what may be driving high-dose prescribing would help reduce such uncertainties. However, evidence has not been synthesized to understand this uncertainty. We, therefore, systematically synthesized the observational evidence to explore factors associated with the prescribing of high-dose opioids in primary care.

## Methods

Our systematic review was designed using the Cochrane Handbook for Systematic Reviews of Interventions [19], adapting it for observational studies. Our review is reported in accordance with the Reporting Checklist for Meta-analyses of Observational Studies (MOOSE) [20] (see Table S1 in Additional file 1).

### Eligibility criteria

We included quantitative observational studies if they (1) were conducted in a primary care setting, defined as the first point of contact for care that can provide continuity of care, including general practice, family medicine, community pharmacy, and dental and optometry services [21]; (2) were conducted in a high-income country as defined by the World Bank [22]; (3) included adults (≥ 18 years old) for whom opioids had been prescribed, stratified by oral morphine equivalents (OME) in milligrams per day (mg/day), with one or more group(s) receiving high doses. We defined high doses of opioids as ≥ 90 OME mg/day as this is the lowest high-dose threshold that guidelines recommend clinicians to avoid [9, 23]; (4) present summarized patient-level data; and (5) reported any factor or factors stratified by high-dose and low-dose opioid groups. We included all languages.

We excluded studies if (1) they were conducted in nursing homes, emergency departments, out-of-hours clinics, outpatient clinics, secondary or tertiary care, or a combination of these settings (i.e., mixed care settings); (2) opioids were measured using a different metric to OME mg/day (e.g., defined daily dose or prescription rate per 1000 population) because OME best reflects prescribing in clinical practice [24]; and (3) the study wholly focused on palliative care, cancer pain, pregnancy or labor pain, opioid-related misuse, overdose and/or death, illicit or non-prescribed opioids, opioid receptor antagonists, and non-community dwelling adults (e.g., prisoners and military personnel).

### Search strategy

We searched MEDLINE (Ovid), Embase (Ovid), and Web of Science Core Collection (excluding Chemical Indexes) from database inception to 5 April 2019. We hand searched forward citations and reference lists of eligible studies. Conference proceedings were also used to identify potentially eligible studies but were not included unless a complete manuscript was published. The search terms and search strategy are available in Table S2 and Table S3 of Additional file 1.

### Study selection

Duplicates were removed after the results of the searches were exported to Endnote X8. Two authors (GCR, TBM) independently screened titles and abstracts for eligibility. Afterwards, two authors (GCR, NJD) individually assessed full texts of studies for eligibility using our predetermined criteria. When necessary, we contacted authors of studies by electronic mail for clarification of inclusion status. We resolved disagreements by consensus or with a third reviewer (KRM, CJH).

### Data extraction

Two authors (GCR, TBM) independently extracted data using a predeveloped data extraction spreadsheet for each eligible study. This included (1) general information and study characteristics: year of publication, geographical location, specific primary care setting, study design, data source, included and excluded populations, and sample size; (2) exposures: high-dose and low-dose thresholds, duration of dose, methods for calculating doses, and morphine equivalent conversion factors; and (3) factors reported by each study (i.e., age, gender, measures of depression). After the list of factors was cross checked, two authors (GCR, NJD) extracted the raw data of each factor with disagreements resolved by consensus.

### Quality and risk of bias assessment

Two authors (GCR, TBM) evaluated the quality of included studies using the National Institute of Health (NIH), National Heart, Lung, and Blood Institute (NHLBI) Quality Assessment Tool for Observational Cohort and Cross-Sectional Studies [25] because it accounts for the assessment of both cohort and cross-sectional studies. This tool evaluates the quality of the research question, reporting of the study population, participation rate, selection of participants, sample size, appropriateness of statistical analyses, timeframe for associations, levels of exposures, ascertainment of the exposure, appropriateness of outcome measures, outcome blinding of assessors, loss to follow-up, and adjustment for confounding, which provide an overall rating of “good,” “fair,” or “poor.” Disagreements were resolved by consensus or discussion with a third author (KRM, CJH). We also extracted data on ethical approvals, participant enrolment incentives, study sponsorship, and declarations of conflicts of interests (COIs). Particular attention was placed on pharmaceutical sponsorship and COIs because of the pharmaceutical industry’s contribution to the opioid crisis [26].

### Data synthesis and analysis

We pooled data using a random effects model where appropriate. For binary outcomes, we reported relative risks (RR) with 95% confidence intervals (CIs). We calculated the number needed to harm (NNT_H_) for binary outcomes that were behavioral in nature (i.e., the use of health services). For continuous data, we calculated the mean differences between high-dose and low-dose groups. When the median, range, and/or interquartile range were reported, we used the method by Wan and colleagues to calculate the sample mean and standard deviation (SD) [27]. When a study included more than two dose groups, we combined the sample means and SDs using Cochrane’s formulae for combining groups [28]. When considerable heterogeneity, defined as *I*^2^ ≥ 75% [29], was found, we conducted sensitivity analyses by removing outliers. We conducted subgroup analyses for studies that differed by quality assessment, design (cross-sectional vs cohort), or main objective. We used Stata software version 16.0 for all analyses.

## Results

**Fig. 1:**
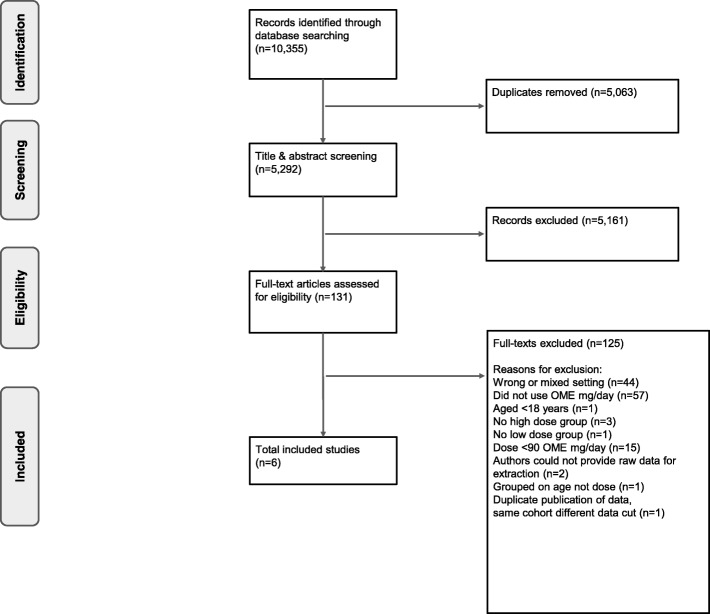
PRISMA flow diagram of the study selection. OME oral morphine equivalent

We screened 5292 titles and abstracts and 131 full-text articles (Fig. 1). Six studies met our eligibility criteria which included more than 4.2 million people taking opioids (*n* = 4,248,119), of whom 3.64% (*n* = 154,749) were using high-dose formulations. The number of included participants was not equally distributed across studies (median 4651.5 participants, range 51–4,046,275). One large cross-sectional study accounts for the majority of participants [30]. This study used a large administrative database from QuintilesIMS that captures 75% of community prescriptions in the United States of America (USA). Three cohort studies used databases that represent large populations—QuintilesIMS in the USA [4], the Clinical Practice Research Datalink (CPRD) in the United Kingdom (UK) [31], and the Kaiser Permanente Northwest virtual data warehouse in the USA [32]. The smaller studies conducted in the USA [33] and Australia [34] actively recruited participants from primary care settings using self-reported measures. Table 1 summarizes the characteristics of participants from included studies.

**Table 1:** Characteristics of participants and included studies ordered by study design and year of publication

Study ID Country [Ref]	Data source	Population	Duration to determine the opioid dose	Dose groups			
				High dose		Low dose	
**Cross-sectional studies**				**mg/day**	***N***	**mg/day**	***N***
Morasco, 2019 USA [33]	Electronic medical records and self-reported measures	51 patients aged 18–70 years receiving opioids for musculoskeletal pain	90-day average	≥ 100	17	5–99	34
Chang, 2018a USA [30]	QuintilesIMS’ LifeLink longitudinal prescription database linked to patient and prescriber files	4,046,275 patients aged ≥ 18 years prescribed an opioid	90-day average	> 100	150,814	≤ 100	3,895,461
**Cohort studies**				**mg/day**	***N***	**mg/day**	***N***
Chang, 2018b USA [4]	QuintilesIMS patient-level administrative claims	191,405 patients aged 18–64 years with at least one prescription opioid claim	90-day average	> 100	2778	≤ 100	188,627
Campbell, 2015 Australia [34]	Telephone interviews, self-reported questionnaire, and medication diary	1085 patients aged ≥ 18 years with CNCP prescribed an opioid for > 6 weeks	1-week average	≥ 91	425	1–90	660
Chapman, 2013 UK [31]	CPRD	4035 patients with CNCP, ≥ 2 office visits and ≥ 1 prescription of fentanyl, hydromorphone, morphine, and/or oxycodone	Dose measured at each visit	> 200	262	≤ 200	3773
Kobus, 2012 USA [32]	Kaiser Permanente Northwest virtual data warehouse	5268 patients aged ≥ 18 years with low-back pain and > 90 consecutive days of opioid use	Last dispensed dose	≥ 100	453	1–99	4815

*CNCP* chronic non-cancer pain, *CPRD* Clinical Practice Research Datalink, *UK* United Kingdom, *USA* United States of America

### Quality and risk of bias assessment

Four studies were rated as “good” quality [4, 30–32], and two studies were rated as “fair” [33, 34]. Studies rated as fair did not adequately justify their sample sizes [33] or did not report whether participants were lost to follow-up and did not control for confounding [34]. No pharmaceutical sponsorship was sought to conduct the included studies. However, eight authors from one included study reported conflicts with two pharmaceutical companies who manufacture opioids [34]. The full assessment is in Table S4 of Additional file 1.

### Meta-analyses of factors associated with high-dose opioids

High-dose opioids were significantly associated with the co-prescription of benzodiazepines (RR 3.27, 95% CI 1.32 to 8.13, *I*^2^ = 99.9%, 4 studies; *n* = 4,248,119). The high degree of heterogeneity is attributable to the two large studies that found participants taking high doses had a five- and eightfold greater risk of being co-prescribed benzodiazepines than participants taking low doses respectively. In a sensitivity analysis removing these studies, high-dose opioids were still significantly associated with the co-prescription of benzodiazepines, with no heterogeneity (RR 1.47, 95% CI 1.37 to 1.59, *I*^2^ = 0%, 2 studies; *n* = 6353, see Figure S1 in Additional file 1).

**Fig. 2:**
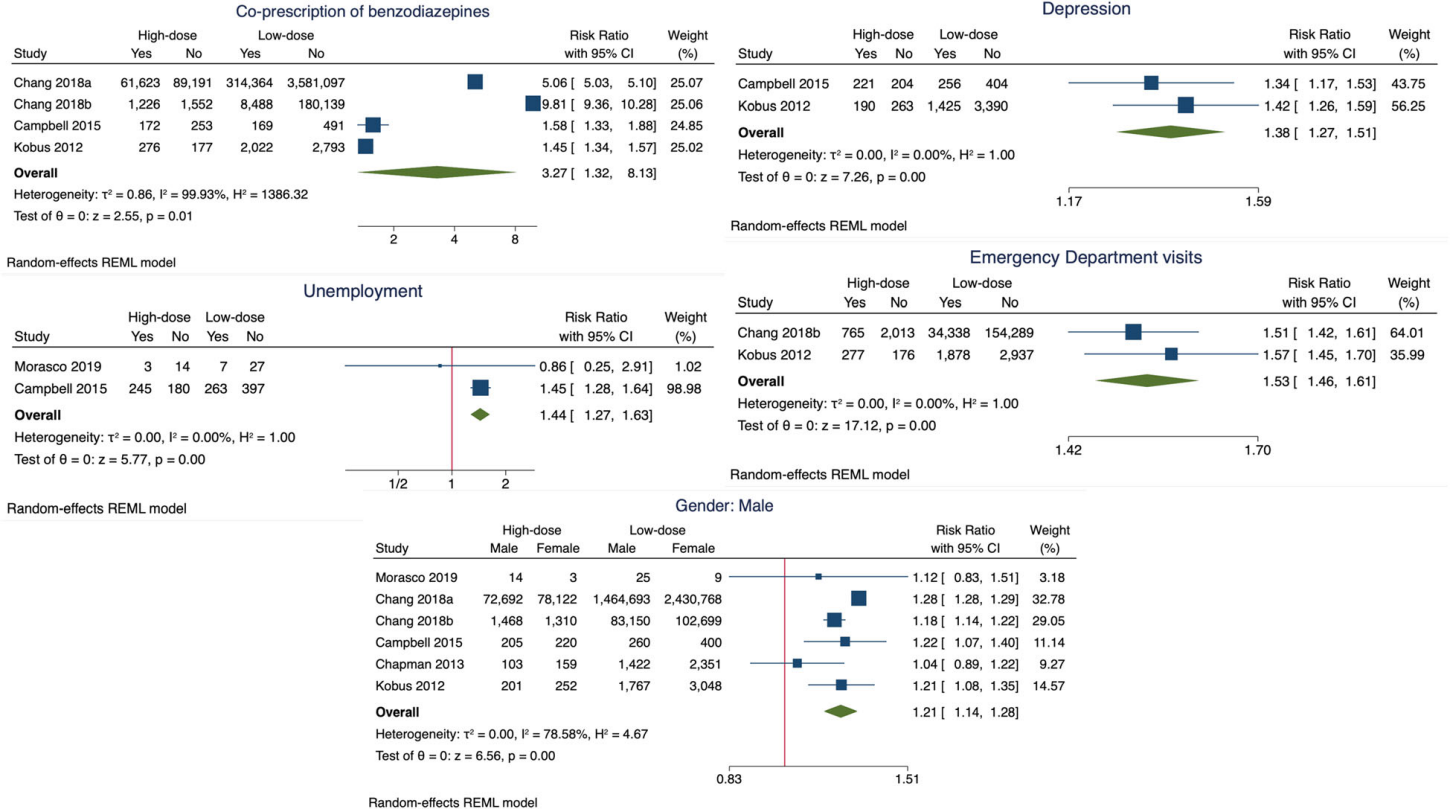
Forrest plots of the factors associated with the prescribing of high-dose opioids in primary care

High-dose opioids were also significantly associated with depression (RR 1.38, 95% CI 1.27 to 1.51, *I*^2^ = 0%, 2 studies; *n* = 6353), emergency department visits (RR 1.53, 95% CI 1.46 to 1.61, *I*^2^ = 0%, 2 studies, *n* = 196,673; NNT_H_ 15, 95% CI 12 to 20), unemployment (RR 1.44, 95% CI 1.27 to 1.63, *I*^2^ = 0%, 2 studies; *n* = 1136), and male gender (RR 1.21, 95% CI 1.14 to 1.28, *I*^2^ = 78.6%, 6 studies; *n* = 4,248,119) when compared to participants taking low doses of opioids (Fig. 2). The sensitivity analysis for gender is presented in Figure S2, Additional file 1.

### Factors associated with high-dose opioids from individual studies

Table 2 presents factors reported by single studies that were significantly associated with high-dose opioids. Diagnosis of pharmaceutical opioid dependence at 12 months was significantly associated with high-dose opioids (RR 3.11, 95% CI 1.61 to 5.98, *n* = 1085). Participants prescribed high-dose opioids were 29 times more likely to have an opioid disorder compared to participants on low doses (RR 28.9, 95% CI 26.3 to 31.8, *n* = 191,405). For every 22 participants on high-dose opioids, one participant reported tampering with their opioids (RR 2.03, 95% CI 1.27 to 3.25, *n* = 108; NNT_H_ 22, 95% CI 13 to 64). Participants on high doses of opioids were more likely to visit a pain clinic within 6 months of entering or leaving the study (RR 2.09, 95% CI 1.73 to 2.51, *n* = 5268; NNT_H_ 8, 95% CI 6 to 12). High-dose opioids were also significantly associated with receiving 50% or more of one’s prescriptions from a high-risk prescriber, defined as a prescriber in the top 5th percentile of opioid volume.

**Table 2:** Factors associated with high-dose opioids reported by individual studies

Study ID [Ref]	Variable	High-dose	Low-dose	RR (95% CI)	NNT_H_ (95% CI)
		Count (%)	Count (%)		
**Sociodemographic characteristics**					
Chang, 2018a [30]	State of residence				
	California	47,446 (31%)	1,416,000 (36%)	0.87 (0.86, 0.87)	NA
	Florida	54,338 (36%)	1,207,982 (31%)	1.16 (1.15, 1.17)	
	Georgia	20,692 (14%)	689,886 (18%)	0.77 (0.76, 0.78)	
	Maryland	12,487 (8%)	250,868 (6%)	1.29 (1.26, 1.31)	
	Washington	15,866 (11%)	330,335 (8%)	1.24 (1.22, 1.26)	
Kobus, 2012 [32]	Insurance coverage				
	Medicare	154 (34%)	1352 (28%)	1.21 (1.06 to 1.39)	NA
	Ethnicity				
	Unknown/declined to answer	64 (14%)	879 (18%)	0.77 (0.61, 0.98)	NA
**Treatment-related factors**					
Campbell, 2015 [34]	Antidepressants	246 (58%)	323 (49%)	1.18 (1.06 to 1.32)	NA
	Type of opioid drug				
	Morphine	86 (20%)	75 (11%)	1.78 (1.34 to 2.37)	NA
	ICD-10 lifetime pharmaceutical opioid dependence	49 (12%)	28 (4%)	2.72 (1.7 to 4.25)	NA
	ICD-10 12-month pharmaceutical opioid dependence	26 (6%)	13 (2%)	3.11 (1.61 to 5.98)	NA
	Prescribed opioid difficulty scale (PODS) intermediate-high (≥ 8)	297 (70%)	367 (56%)	1.26 (1.15 to 1.38)	NA
	Past 3-month tampering	38 (9%)	29 (4%)	2.03 (1.27 to 3.25)	22 (13 to 64)
	Past 3-month different drug route	7 (2%)	1 (0.2%)	10.87 (1.34 to 88.04)	NA
Kobus, 2012 [32]	Long-acting opioids	400 (88%)	1637 (34%)	2.60 (2.47, 2.74)	NA
**Substance use**					
Chang, 2018b [4]	Opioid disorders	530 (19%)	1243 (1%)	28.95 (26.34, 31.82)	NA
Campbell, 2015 [34]	Illicit drug use past 12 months	71 (17%)	67 (10%)	11.03 (5.75 to 21.14)	NA
Kobus, 2012 [32]	Substance use disorder	141 (31%)	1151 (24%)	1.30 (1.13 to 1.51)	NA
**Clinical factors**					
Campbell, 2015 [34]	Back or neck problems	344 (81%)	484 (73%)	1.10 (1.03 to 1.18)	NA
	Frequent/severe headaches	134 (32%)	170 (26%)	1.22 (1.01 to 1.48)	NA
**Healthcare utilization**					
Chang, 2018a [30]	Opioids from ≥ 4 unique prescribers and pharmacies over 90 days	1176 (0.78%)	1948 (0.05%)	15.6 (14.51 to 16.76)	137 (129 to 145)
Chang, 2018b [4]	> 1 Hospitalizations				
	Concurrent 2012	443 (16%)	17,061 (9%)	1.76 (1.62, 1.92)	14 (12 to 18)
	Prospective 2013	396 (14%)	11,110 (6%)	2.42 (2.21, 2.66)	12 (10 to 14)
Kobus, 2012 [32]	Any pain clinic visits 6 months before/after index date	104 (23%)	530 (11%)	2.09 (1.73 to 2.51)	8 (6 to 12)
	Filled opioid prescription 5 days after emergency department visit	285 (63%)	2696 (56%)	1.12 (1.04 to 1.21)	14 (9 to 46)
**Mental health**					
Kobus, 2012 [32]	Posttraumatic stress disorder diagnostic code 309.81	20 (4%)	96 (2%)	2.21 (1.38 to 3.55)	NA
**Prescribers**					
		**Mean (%)**	**Mean (%)**	**RR (95% CI)**	
Chang, 2018a [30]	Proportion of prescriptions from high-risk* prescribers	122,159 (81%)	973,865 (25%)	3.24 (3.23 to 3.25)	NA
		**Count (%)**	**Count (%)**	**RR (95% CI)**	
	100% of opioid prescriptions from high-risk* prescribers	77,217 (51%)	572,633 (15%)	3.48 (3.46 to 3.50)	NA
	50–99% of prescriptions from high-risk* prescribers	51,277 (34%)	471,351 (12%)	2.81 (2.79 to 2.83)	NA

*CI* confidence interval, *ICD-10* international classification of diseases 10th revision, *NNT*_*H*_ number needed to harm, *RR* relative risk, *high-risk prescribers were defined as those in the top 5th percentile of opioid volume

### Factors not associated with high-dose opioids

Age (mean difference − 1.94, 95% CI − 4.93 to 1.04, *I*^2^ = 94.4%, 4 studies; *n* = 4,052,679), Caucasian ethnicity (RR 1.09, 95% CI 0.98 to 1.20, *I*^2^ = 19.5%, 2 studies; *n* = 5319), and anxiety (RR 1.44, 95% CI 0.87 to 2.38, *I*^2^ = 90.71%, 2 studies; *n* = 6353) were not associated with high-dose opioids (see Figure S3, Additional file 1). There were a number of factors not associated with high-dose opioids from individual studies including the use of over-the-counter analgesics (RR 0.95, 95% CI 0.86 to 1.04, *n* = 1085), a BMI greater than or equal to 30 (RR 1.05, 95% CI 0.95 to 1.15, *n* = 5268), and arthritis or rheumatism pain (RR 0.94, 95% CI 0.85 to 1.03, *n* = 1085) (see Table S5, Additional file 1). All factors reported by included studies are summarized in Table S6, Additional file 1. Raw data extracted from included studies is available in Additional file 2.

## Discussion

We pooled patient-level data from over four million participants taking opioids and found that high doses of opioids are associated with the co-prescription of benzodiazepines, depression, more visits to emergency departments, unemployment, and male gender. We conclude that people taking opioids in high doses are at a greater risk of harm which warrants closer management in primary care.

### Comparison with existing literature

Our review is the first to synthesize factors associated with high-dose opioids in primary care, so comparison with existing systematic reviews is not possible. Others have conducted a narrative review on the association between opioid dose and the risk of misuse, abuse, addiction, overdose, and death which concluded that increasing opioid dose is associated with an increased risk of serious harm [35]. Quinlan and colleagues conducted a review on the risk factors for opioid dependence following surgery and proposed strategies to mitigate dependence including the avoidance of repeat opioid prescriptions postoperatively [36]. Our findings align with the notion of “adverse selection,” whereby the riskiest drugs and doses are prescribed to those who will most probably be harmed by them [37, 38]. Thus, biases in prescribers’ clinical decision-making may underlie some of the observed associations.

Anxiety was not associated with high doses of opioids despite people taking high doses to be three times more likely to have benzodiazepines co-prescribed. Campbell et al. [34] found one fifth of people taking both high- and low-dose opioids to have moderate to severe anxiety while Kobus et al. [32] found those on high doses to be twice as likely to have an ICD-9 code for anxiety than people on low doses. This may be explained by comorbidities, such as insomnia, and the severity of anxiety reported by participants. Non-pharmacological treatments for anxiety are the preferred first-line treatment for people on opioids [39].

### Implications for practice and policy

Our findings provide priority areas that clinicians, policymakers, medicine regulators, and commissioners can use in their plight to manage the growing opioid crisis. Monitoring the prescribing of opioids via clinical dashboards or electronic medical records has improved adherence to guidelines, reduced opioid doses, and improved physicians’ knowledge and attitudes towards managing people on opioids in primary care [40–42]. Strategies that promote the safe prescribing of opioids and enable prescribers to effectively manage factors such as benzodiazepine co-prescription and depression are needed.

The concurrent use of benzodiazepines with opioids increases the risk of overdose deaths and the use of health services compared with taking opioids alone [43, 44]. Primary care prescribers should thus carefully consider whether to continue prescribing this combination of drugs and, when the combination is deemed necessary, should discourage continuous benzodiazepine treatment lasting two or more months and marked dose increases [45]. Efforts have been made to audit the number of people taking high doses of opioids in primary care [46, 47]. Providers should extend this audit to include co-prescribed benzodiazepines using real-time audit and feedback tools [48].

The management and treatment of depression is another priority area highlighted by our findings. Although the causal mechanism between opioids and depression cannot be elucidated from our review, others have found new onset depression is associated with the duration of opioid use but not dose [49]. The addition of depression to an already long list of harms invites reconsideration for the merits of prescribing high-dose opioids. Primary care providers should exercise caution, consider a gradual taper, and offer close medical supervision for people with depression who are taking high-dose opioids [50].

For every 15 participants on high-dose opioids, one will present to the emergency department. This finding highlights the impact high-dose opioid use has on healthcare systems. Although high-income countries have different healthcare systems, reducing the prescribing of high-dose opioids has potential for cost savings. In the USA, it is estimated that the opioid crisis has cost more than $72.4 billion [51]. Various regulations have been enacted to reduce high-risk prescribing practices including mandatory Prescription Drug Monitoring Programs, caps on opioid prescribing which limit the dose and/or duration of prescriptions, and pill mill laws to prevent nonmedical opioid prescribing. However, the effectiveness of such USA state laws is currently under investigation [52]. In England, if every general practice prescribed high-dose opioids at the same rate as the lowest decile of practices, a cost saving of £24.8 million and 543,000 fewer high-dose opioid prescriptions could be achieved in 6 months [5]. Thus, investing in resources and programs tailored to people taking opioids in primary care such as the service evaluated by Scott et al. [53] could reduce the need for people on high doses to visit emergency departments.

Male gender and unemployment were statistically associated with high-dose opioids; however, sociodemographic factors may not have much clinical utility. Alternatively, sociodemographic factors may be useful proxies to help identify people taking high doses and amenable to therapeutic interventions.

### Strengths and limitations of the study

Despite the strengths of our systematic review and unique focus on high-dose opioids, this review has several limitations. Firstly, the inherent limitations and complexities of using observational evidence impacts the quality and availability of data. The majority of included studies were conducted in the USA. Thus, our findings are less generalizable to parts of Europe and Oceania and are not applicable to low- and middle-income countries where access to opioids is inadequate [54]. Reported factors vary considerably across studies and so few studies were eligible for meta-analysis. It is not possible to determine the causal or temporal relationship between factors associated with high-dose opioids. It is therefore unclear whether factors such as depression were present before the participant was titrated to high doses and, if so, whether taking opioids in high doses worsened pre-existing depression.

Secondly, few studies were included in our review because most observational studies on the prescribing of high-dose opioids use population-level prescribing data. Summarized patient-level data is a critical eligibility criterion and strength of our review. Despite this, two large studies [4, 30] did not report data on the comorbidities or the indication for high doses and thus the proportion of people taking opioids for palliative care or cancer pain is unclear in these studies. Observational studies on the prescribing of high-dose opioids should report the indication for prescribing when available.

Thirdly, there is no standard or consensus definition of what constitutes high dose, and there is substantial heterogeneity in the proportion of participants on high doses, and the methods and conversion factors used to calculate OME. For the purposes of this review, we defined high dose as ≥ 90 OME mg/day and low dose as < 90 OME mg/day, based on well-established guidelines [9, 23]. An individual’s daily dose may not consistently sit within our defined high-dose and low-dose thresholds. For example, a patient may experience less pain on one day and not consume their complete daily dose. In contrast, when pain is bad, they may exceed their daily dose. Thus, actual exposure may be different from what is prescribed or dispensed. Raw data would be needed from study authors to conduct subgroup analyses on alternative definitions for “high-dose” opioid use. The impact of opioid tolerance, comorbidities, polypharmacy, genetics, and other individual characteristics are barriers to standardizing a definition and advocating best practices for safe and effective prescribing of opioids at high doses.

### Future research

A coordinated international effort is needed to understand unique country-specific drivers of high-dose opioid use. Future research should prospectively examine patient-level data on the prescribing of high-dose opioids in primary care to control for confounding and to understand the relationships between associated factors. Prescribers’ clinical decision-making regarding dose escalation or reduction of opioids, as well as the benefits and harms of this, warrants further investigation. Improving the use of diagnostic codes in people with chronic pain and reporting indications for prescribing high-dose opioids in primary care is needed. There is also a need for standardizing methods and core outcomes in studies investigating opioids to facilitate evidence synthesis and understand differences between and within populations.

## Conclusions

Our findings affirm that people taking high-dose opioids in high-income primary care settings are at greater risk of harm. The use of benzodiazepines, treatment of depression, and frequent visits to emergency departments are priority areas that can be taken into account when selecting, identifying, and managing people and services for people taking high doses of opioids in primary care. Standardizing the reporting of all outcomes and promoting the sharing of data from observational studies would help identify all potential factors associated with the prescribing of high-dose opioids in primary care. While we recognize the limitations of observational evidence, the absence of data on comorbidities and indications for opioids, the complexities of chronic pain, and the clinical challenges of managing pain, our findings illustrate that more resources in primary care are warranted to support people taking high doses of opioids.

## Supplementary information

**Additional file 1.** Supplementary Materials. This contains Tables S1-S6 and Figure S1-S3.

**Additional file 2.** Data File. This file contains all raw data (factors) extracted from the six included studies.

## Data Availability

All data analyzed during the study are included in this publication article and its supplementary information files.
