# Supplementary material for: Factors associated with the prescribing of high-dose opioids in primary care: a systematic review and meta-analysis
Source: BMC Med. 2020 Mar 30;18:68. doi: 10.1186/s12916-020-01528-7 (PMC7104520; doi:10.1186/s12916-020-01528-7)
Supplement: Supplementary file 1 — Additional file 1. Supplementary Materials. This contains Tables S1-S6 and Figure S1-S3. [file 12916_2020_1528_MOESM1_ESM.pdf]

Additional File 1: Supplementary Materials

Table S1. MOOSE Checklist for Meta-analyses of Observational Studies.

Table S2. International brand names for opioids collated using the UpToDate database.

Table S3. MEDLINE search strategy and results.

Table S4. Quality assessment of included studies, using the National Institute of Health, National Heart, Lung & Blood Institute Quality Assessment Tool for Observational Cohort & Cross-Sectional Studies with additional data.

Figure S1. Forest plot of the sensitivity analysis conducted for benzodiazepine co-prescriptions.

Figure S2. Forest plot of the sensitivity analysis conducted for gender.

Figure S3. Forest plots of factors not associated with the prescribing of high-dose opioids in primary care.

Table S5. Factors not associated with the prescribing of high-dose opioids reported by individual studies.

Table S6. All factors reported by included studies.

Table S1. MOOSE Checklist for Meta-analyses of Observational Studies.

Item No	Recommendation	Line numbers	Text
Reporting of background should include			
1	Problem definition	105-107	"understanding who is taking high doses and what may be driving high-dose prescribing would help reduce such uncertainties. However, evidence has not been synthesized to understand this uncertainty."
2	Hypothesis statement	-	-
3	Description of study outcome(s)	108	"factors"
4	Type of exposure or intervention used	108	"high-dose opioids"
5	Type of study designs used	108	"observational evidence"
6	Study population	109	"primary care"
Reporting of search strategy should include			
7	Qualifications of searchers (eg, librarians and investigators)	Table S2	"We developed the search strategy in consultation with an information specialist."
8	Search strategy, including time period included in the synthesis and key words	146<br>Table S2	"The search terms and search strategy are available in S2 Table and S3 Table." "Search terms included "opioid*", "prescri*", "factor*", "primary healthcare" and variations of these, including specific drug and brand names of opioids (see Table S2). The search strategy was initially developed for Medline as presented in S3 Table, then adapted for Embase and Web of Science databases."
9	Effort to include all available studies, including contact with authors	128<br>153-154	"We included all languages."<br>"we contacted authors of studies by electronic mail"
10	Databases and registries searched	142	"Medline (Ovid), Embase (Ovid), and Web of Science Core Collection"
11	Search software used, name and version, including special features used	150<br>194-195	"Endnote X8"<br>"Stata software version 16.0"
12	Use of hand searching	143-144	"We hand searched forward citations and reference lists of eligible studies"
13	List of citations located and those excluded, including justification	119-139<br>Figure 1	"We included …"<br>"We excluded studies if…"
14	Method of addressing articles published in languages other than English	128	"We included all languages."
15	Method of handling abstracts and unpublished studies	144-147	"Conference proceedings were also used to identify potentially eligible studies but were not included unless a complete manuscript was published."
16	Description of any contact with authors	153-154	"we contacted authors of studies by electronic mail for clarification of inclusion status."

Reporting of methods should include			
17	Description of relevance or appropriateness of studies assembled for assessing the hypothesis to be tested	119-139	"We included …"<br>"We excluded studies if…"
18	Rationale for the selection and coding of data	158-165	"extracted data using a predeveloped data extraction spreadsheet…"
19	Documentation of how data were classified and coded	Not applicable	Not applicable
20	Assessment of confounding appropriate	175	"adjustment for confounding"
21	Assessment of study quality, including blinding of quality assessors, stratification or regression on possible predictors of study results	168-180	"Quality and risk of bias assessment…"
22	Assessment of heterogeneity	191-193	"When considerable heterogeneity, defined as $I^2 \geq 75\%$..."
23	Description of statistical methods in sufficient detail to be replicated	182-195	"Data synthesis and analysis…"
24	Provision of appropriate tables and graphics	Table 1-2 Figures 1-2	
**Reporting of results should include**			
25	Graphic summarizing individual study estimates and overall estimate	Figure 2, Figures S1-S3	-
26	Table giving descriptive information for each study included	Table 1	-
27	Results of sensitivity testing (eg, subgroup analysis)	Figure S1 & S2	-
28	Indication of statistical uncertainty of findings	219-224	Quality and risk of bias & the reporting of CIs
**Reporting of discussion should include**			
29	Quantitative assessment of bias (e.g. publication bias)	363-406	"…inherent limitations and complexities of using observational evidence impacts the quality and availability of data."<br>"…two large studies did not report data on the co-morbidities or the indication for high doses…"
30	Justification for exclusions	380-381	"few studies were included in our review because most observational studies on the prescribing of high-dose opioids use population-level prescribing data"
31	Assessment of quality of included studies	-	In results, lines 219-224
**Reporting of conclusions should include**			
32	Consideration of alternative explanations for observed results	-	-
33	Generalization of the conclusions	420	"high-income primary care settings"
34	Guidelines for future research	424-426	"Standardizing the reporting of all outcomes and promoting the sharing of data from observational studies"
35	Disclosure of funding source	532-537	"Funding: …"

Search strategy

We developed the search strategy in consultation with an information specialist. Search terms included "opioid*", "prescri*", "factor*", "primary healthcare" and variations of these, including specific drug and brand names of opioids (see Table S2). The search strategy was initially developed for Medline as presented in Table S3, then adapted for Embase and Web of Science databases. The first search was run on 12 February 2018. We updated this search in all three databases on 5 April 2019.

Table S2. International brand names for opioids collated using the UpToDate database

Drug name	Brand names	Countries	# of countries	Included or excluded
**Codeine**	N/A	N/A	N/A	Included
**Morphine**	MS Contin	AU, BE, CN, IT, LU, NL, VE, KR	8	Included
	MST Continus	AE, AR, BF, BG, BH, BJ, CI, CY, CZ, EE, EG, ES, ET, GB, GH, GM, GN, HK, HR, ID, IE, IQ, IR, JO, KE, KW, LB, LR, LY, MA, ML, MR, MT, MU, MW, MY, NE, NG, OM, PH, PK, PL, QA, RO, SA, SC, SD, SG, SK, SL, SN, SY, TH, TN, TR, TW, TZ, UG, YE, ZA, ZM, ZW	61	Included
	Oramorph	BE, ES, FR, GB, IE, LU, PT, SE	8	Included
	Sevredol	AU, CH, CZ, HR, IE, NZ, RO, SI, SK, TR	10	Included
**Hydro-morphone**	Himop	CR, DO, GT, HN, NI, PA, SV	7	Included
	Jurnista	AT, AU, CR, CZ, DE, DK, DO, EE, ES, GT, HN, HU, ID, IT, MX, NI, NZ, PA, PH, PT, SA, SG, SI, SV, ZW	25	Included
	Palladon*	CH, DK, FI, IS, NL, NO, SE, SI, SE, BE, CZ, EE, ES, GB, GR, HN, IE, IL, LU, PT	20	Included

	Liberaxim	CR, DO, GT, HN, MX, NI, PA, SV	8	Excluded
**Nicomorphine**	N/A	N/A	N/A	Included
**Oxycodone**	Oxycontin	AR, AT, BR, CH, CL, CN, CO, CR, CY, CZ, DK, DO, EC, EE, ES, FI, GB, GT, HK, HN, IE, IL, IT, MY, NI, NL, NO, NZ, PA, PE, PH, PL, PT, SE, SG, SV, VE, HR, HU, IS, LU, RO, SI, SK, TR, VN	45	Included
	Oxynorm	AT, AU, BE, CH, CY, DK, ES, FI, FR, GB, HK, IE, IS, MY, NO, NZ, PH, SE, SG, TR, JP, SG,	22	Included
**Dihydro-codeine**	N/A	N/A	N/A	Included
**Papaveretum**	N/A	N/A	N/A	Included
**Ketobemidone**	N/A	N/A	N/A	Included
**Pethidine**	Meperidine	N/A	N/A	Included
	Dolosal	BR, CR, DO, GT, HN, NI, PA, SV	8	Included
**Fentanyl**	Abstral	BM, ES, GB, HR, IE, PH, QA, TR	8	Included
	Actiq	AU, CH, DE, DK, ES, FI, FR, GB, IE, IL, KR, PT, SE	13	Included
	Durogesic	AE, AU, BH, CN, CO, CY, EG, ES, ID, IN, JO, KW, LB, LK, MX, NZ, PH, PK, PY, QA, SA, SG, TH, VN	24	Included
	Instanyl	AT, BE, CZ, DE, DK, EE, ES, FR, HR, IS, LT, LU, MT, NL, PL, SE, SI, SK	18	Included
	Ionsys	AT, BE, BG, CH, CZ, DE, DK, EE, FI, FR, GB, GR, HN, IE, IT, MT, NL, NO, PT, RU, SE, SK, TR	23	Included

	Sublimaze	AR, AU, GB, IE, NZ, PH, ZA	7	Included
**Dextro-moramide**	N/A	N/A	N/A	Included
**Piritramide**	N/A	N/A	N/A	Included
**Dextro-propoxyphene**	N/A	N/A	N/A	Included
**Bezitramide**	N/A	N/A	N/A	Included
**Pentazocine**	Fortral	AT, AU, BG, DE, DK, GB, HR, IE, NZ, PL	10	Included
	Fortwin	BF, BJ, CI, ET, GH, GM, GN, IN, KE, LR, MA, ML, MR, MU, MW, NE, NG, SC, SD, SL, SN, TN, TZ, UG, ZM, ZW	29	Included
	Sosegon	AE, BF, BH, BJ, CI, CY, EC, EG, ET, GH, GM, GN, IL, IQ, IR, JO, JP, KE, KW, LB, LR, LY, MA, ML, MR, MU, MW, NE, NG, OM, PK, PT, QA, SA, SC, SD, SL, SN, SY, TN, TZ, UG, YE, ZM, ZW	42	Included
**Phenazocine**	N/A	N/A	N/A	Included
**Bupren-orphine**	Norspan patch	AU, DE, DK, EE, FI, HK, KR, NO, NZ, PH	10	Included
	Subutex	AE, AT, AU, BE, BG, CH, CZ, DE, DK, FR, GR, HR, ID, IE, IL, IS, LU, LV, MT, NO, PT, QA, SE, TW	23	Included
	Temgesic	AE, AT, BE, BF, BH, BJ, BR, CH, CI, CY, DE, DK, EE, EG, ET, FI, FR, GB, GH, GM, GN, GR, HK, IQ, IR, IT, JO, KE, KW, LB, LR, LU, LY, MA, ML, MR, MU, MW, MX, NE, NG, NL, NO, NZ, OM, PK, QA, RU, SA, SC, SD, SE, SG, SK, SL, SN, SY, TN, TR, TW, TZ, UG, YE, ZA, ZM, ZW	66	Included

	Transtec	BE, CH, CL, CO, DE, DK, EC, ES, GB, HN, HR, HU, IE, IT, MX, NL, NO, PE, PL, PT, SK	21	Included
**Butorphanol**	N/A	N/A	N/A	Included
**Nalbuphine**	Bufigen	CR, DO, GT, HN, MX, NI, PA, SV	8	Excluded
	Nalcryn	CR, DO, GT, HN, MX, NI, PA, SV	8	Excluded
	Nubain	AE, AT, BF, BH, BJ, BR, CI, CY, CZ, DE, EE, EG, ET, GB, GH, GM, GN, GR, HU, IE, IL, IQ, IR, JO, KE, KW, LB, LR, LY, MA, ML, MR, MU, MW, MY, NE, NG, NL, OM, PH, PK, PL, QA, SA, SC, SD, SI, SL, SN, SY, TN, TZ, UG, VE, YE, ZA, ZM, ZW	57	Included
**Methadone**	Amidone	CR, DO, GT, HN, NI, PA, SV	7	Included
	Rubidexol	CR, DO, GT, HN, MX, NI, PA, SV	8	Excluded
**Tramadol**	Adamon	AR, CR, DO, GT, HN, NI, PA, PY, SV	9	Included
	Bongesic	CR, DO, GT, HN, NI, PA, SV	7	Excluded
	Mabron	AE, BH, CY, EG, ET, IQ, IR, JO, KW, LB, LV, LY, MY, OM, QA, SA, SG, SY, YE	20	Excluded
	Trabilin	BB, BM, BS, BZ, CR, DO, GT, GY, HN, JM, NI, PA, SR, SV, TT	15	Excluded
	Tradolan	AE, BH, CY, EG, IQ, IR, JO, KW, LB, LY, OM, QA, SA, SE, SY, YE	16	Excluded

	Tramal	AE, AT, AU, BF, BH, BJ, CH, CI, CN, CO, CR, CU, CY, CZ, DE, DO, EC, EE, EG, ET, FI, GH, GM, GN, GR, GT, HN, HR, IQ, IR, JO, JP, KE, KW, LB, LR, LU, LY, MA, ML, MR, MT, MU, MW, NE, NG, NI, NL, NZ, OM, PA, PE, PH, PK, PL, PT, QA, RU, SA, SC, SD, SK, SL, SN, SV, SY, TH, TN, TW, TZ, UG, VE, YE, ZA, ZM, ZW	72	Included
	Tramazac	BF, BJ, CI, ET, GH, GM, GN, IN, KE, LR, MA, ML, MR, MU, MW, NE, NG, SC, SD, SL, SN, TH, TN, TZ, UG, ZM, ZW	27	Included
**Tilidine**	N/A	N/A	N/A	Included
**Dezocine**	N/A	N/A	N/A	Included
**Meptazinol**	N/A	N/A	N/A	Included
**Tapentadol**	Palexia*	AT, BG, CH, CR, CZ, DK, DO, EE, ES, FI, FR, GB, GT, HN, HR, IE, IL, LT, LU, LV, MT, NI, NL, NO, PA, PL, PT, SI, SK, SV, TR, AT, BG, CR, DE, DO, EE, ES, GT, HN, HR, LT, LV, MT, NI, NL, PA, PL, PT, RO, SK, SV	52	Included

Table S3. MEDLINE strategy and results.

Search #	Search terms	# of results
**1**	exp Analgesics, Opioid/	109489
**2**	exp Narcotics/	117473
**3**	1 or 2	117473
**4**	opioid*.ti,ab.	79690
**5**	narcotic*.ti,ab.	14579
**6**	"opioid analgesic*".ti,ab.	4475
**7**	opiate*.ti,ab.	24064
**8**	opium.ti,ab.	2439
**9**	4 or 5 or 6 or 7 or 8	112098
**10**	codeine*.ti,ab.	4774
**11**	morphine*.ti,ab.	48019
**12**	("MS Contin" or "MST Continus" or Oramorph or Sevredol).ti,ab.	118
**13**	Papaveretum.ti,ab.	138
**14**	Ketobemidone.ti,ab.	134
**15**	Dextromoramide.ti,ab.	177
**16**	Hydromorphone.ti,ab.	1466
**17**	(Himop or Jurnista or Palladon*).ti,ab.	14
**18**	Piritramide.ti,ab.	353
**19**	Dextropropoxyphene*.ti,ab.	530
**20**	Oxycodone.ti,ab.	2989
**21**	(Oxycontin or Oxynorm).ti,ab.	240
**22**	Dihydrocodeine.ti,ab.	433
**23**	(Meperidine or Pethidine*).ti,ab.	4850
**24**	Nicomorphine.ti,ab.	43
**25**	Dolosal.ti,ab.	20
**26**	Fentanyl.ti,ab.	17437

**27**	Abstral.ti,ab.	7
**28**	Actiq.ti,ab.	26
**29**	Durogesic.ti,ab.	44
**30**	(Instanyl or Ionsys or Sublimaze).ti,ab.	43
**31**	Methadone.ti,ab.	12800
**32**	Amidone.ti,ab.	44
**33**	Pentazocine.ti,ab.	2333
**34**	(Fortral or Fortwin or Sosegon).ti,ab.	52
**35**	Bezitramide.ti,ab.	20
**36**	Phenazocine.ti,ab.	70
**37**	Buprenorphine.ti,ab.	6016
**38**	Norspan*.ti,ab.	4
**39**	Subutex.ti,ab.	94
**40**	(Temgesic or Transtec).ti,ab.	70
**41**	Butorphanol.ti,ab.	1434
**42**	Nalbuphine.ti,ab.	885
**43**	Nubain.ti,ab.	44
**44**	Tramadol.ti,ab.	4608
**45**	Adamon.ti,ab.	9
**46**	(Tramal or Tramazac).ti,ab.	98
**47**	Tilidine.ti,ab.	133
**48**	Tapentadol.ti,ab.	418
**49**	Palexia*.ti,ab.	10
**50**	Dezocine.ti,ab.	142
**51**	meptazinol.ti,ab.	219
**52**	10 or 11 or 12 or 13 or 14 or 15 or 16 or 17 or 18 or 19 or 20 or 21 or 22 or 23 or 24 or 25 or 26 or 27 or 28 or 29 or 30 or 31 or 32 or 33 or 34 or 35 or 36 or 37 or 38 or 39 or 40 or 41 or 42 or 43 or 44 or 45 or 46 or 47 or 48 or 49 or 50 or 51	92514
**53**	3 or 9 or 52	196757

**54**	exp INAPPROPRIATE PRESCRIBING/ or exp Drug Prescriptions/ or exp Drug Utilization/ or exp Practice Patterns, Physicians'/	98731
**55**	exp Potentially Inappropriate Medication List/	279
**56**	exp Prescription Drugs/	5235
**57**	exp Prescription Drug Misuse/	11706
**58**	exp Medical Overuse/ or exp Deprescriptions/ or exp Prescriptions/	39044
**59**	exp Prescription Drug Overuse/ or exp Drug Misuse/ or exp Self Medication/	16227
**60**	exp Behind-the-Counter Drugs/ or exp Nonprescription Drugs/	5885
**61**	prescri*.ti,ab.	195505
**62**	Utiliz*.ti,ab.	514119
**63**	utilis*.ti,ab.	44114
**64**	dispens*.ti,ab.	35225
**65**	54 or 55 or 56 or 57 or 58 or 59 or 60 or 61 or 62 or 63 or 64	852797
**66**	53 and 65	19678
**67**	exp Primary Health Care/	145132
**68**	exp Ambulatory Care Facilities/	52697
**69**	exp Physicians, Primary Care/	2888
**70**	exp Outpatient Clinics, Hospital/	16767
**71**	exp Ambulatory Care/	51044
**72**	exp Emergency Service, Hospital/ or exp Family Practice/ or exp General Practice/	142600
**73**	exp General Practice, Dental/	4727
**74**	exp Office Visits/	6681
**75**	exp Physicians, Family/ or exp General Practitioners/	22706
**76**	exp Emergency Medical Services/	128929
**77**	exp Pharmacies/ or exp Community Pharmacy Services/	11288
**78**	exp Community Health Services/ or exp Community Medicine/ or exp Community Health Nursing/ or exp Community Health Centers/	299456

**79**	exp Community Dentistry/	1213
**80**	exp Home Nursing/ or exp Residential Facilities/	58938
**81**	(ambulatory adj5 (department? or dept* or ward? or room? or unit? or service? or care or setting? or facilit*)).ti,ab.	17870
**82**	((general or family) adj2 (practi* or physician? or doctor?)).ti,ab.	112376
**83**	((primary* adj3 (care or health*)) or community or communities or population).ti,ab.	1775651
**84**	(clinic? or office or visit? or "health centre" or "health center" or "medical centre" or "medical center").ti,ab.	560228
**85**	("out of hours" or ooh or "after hours").ti,ab.	4235
**86**	(emergency adj5 (department? or dept* or ward? or room? or unit? or service? or care or setting? or facilit*)).ti,ab.	127109
**87**	exp Housing for the Elderly/ or exp Assisted Living Facilities/ or exp Home Care Services/ or exp Homes for the Aged/	60450
**88**	exp INSTITUTIONALIZATION/ or exp Long-Term Care/	32735
**89**	((nursing or residential or longterm or long-term or institutional) adj2 home).ti,ab.	23609
**90**	(((residential or longterm or long-term) adj2 care) or facilit*).ti,ab.	624294
**91**	exp Home Care Services/	45434
**92**	((home or domiciliary) adj (visit* or call*)).ti,ab.	8212
**93**	((refill or repeat) adj prescri*).ti,ab.	461
**94**	67 or 68 or 69 or 70 or 71 or 72 or 73 or 74 or 75 or 76 or 77 or 78 or 79 or 80 or 81 or 82 or 83 or 84 or 85 or 86 or 87 or 88 or 89 or 90 or 91 or 92 or 93	3285822
**95**	66 and 94	7761
**96**	predict*.ti,ab.	1419106
**97**	characteristic*.ti,ab.	1259013
**98**	varian*.ti,ab.	480043
**99**	variat*.ti,ab.	649635
**100**	factor*.ti,ab.	3073031
**101**	96 or 97 or 98 or 99 or 100	5779446
**102**	95 and 101	2612

Table S4. Quality assessment of included studies, using the National Institute of Health, National Heart, Lung & Blood Institute Quality Assessment Tool for Observational Cohort & Cross-Sectional Studies with additional data to assess risk of bias.

	NIH NHLBI Quality Assessment Tool for Observational Cohort & Cross-Sectional Studies																	Additional data					
**Study ID**	**1**	**2**	**3**	**4**	**5a**	**5b**	**5c**	**6**	**7**	**8**	**9**	**10**	**11**	**12**	**13**	**14**	**Quality rating**	**Ethical approval**	**Enrolment incentives**	**Study sponsor**	**Pharmaceutical sponsorship**	**COI declared**	**Pharmaceutical COI**
**Morasco, 2019**	✓	✓	✓	✓	✓	✗	✗	✓	✓	✓	✓	✗	✓	✗	NA	✓	**Fair**	NR	NR	✓	✗	✓	✗
**Chang, 2018a**	✓	✓	NA	✓	NA	✗	✓	✓	✓	✓	✓	✗	✓	✗	NA	✓	**Good**	NR	NA	NR	✗	✓	✗
**Chang, 2018b**	✓	✓	NA	✓	NA	✗	✓	✓	✓	✓	✓	✗	✓	✗	NA	✓	**Good**	Deemed not required	NA	NR	✗	✓	✗
**Campbell, 2015**	✓	✓	✓	✓	✓	✗	✓	✓	✓	✓	✓	✗	✓	✗	NR	✗	**Fair**	✓	$20 AUD to Pharmacists for referrals	✓	✗	✓	Reckitt Benckiser & Mundipharma
**Chapman, 2013**	✓	✓	NA	✓	✓	✗	✓	✓	✓	✓	✓	✓	✓	✗	NA	✓	**Good**	✓	NA	✓	✗	None to report	✗
**Kobus, 2012**	✓	✓	NA	✓	✓	✗	✓	✓	✓	✓	✓	✗	✓	✗	NA	✓	**Good**	✓	NA	✓	✗	None to report	✗

✓: yes; ✗: no; COI: conflict of interest; NA: not applicable; NR: not reported; 5a refers to sample size justification, 5b power description and 5c a measure of variance and effect estimates.

Figure S1. Forest plot of the sensitivity analysis conducted for benzodiazepine co-prescriptions

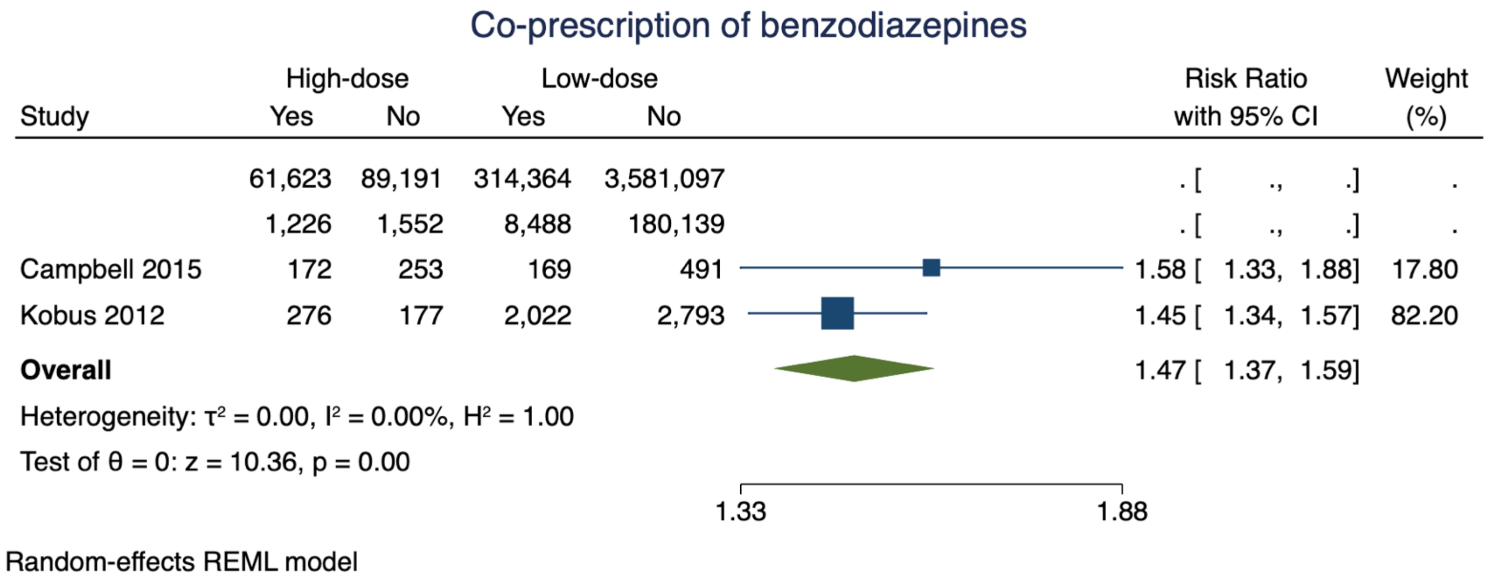

Figure S2. Forest plot of the sensitivity analysis conducted for gender

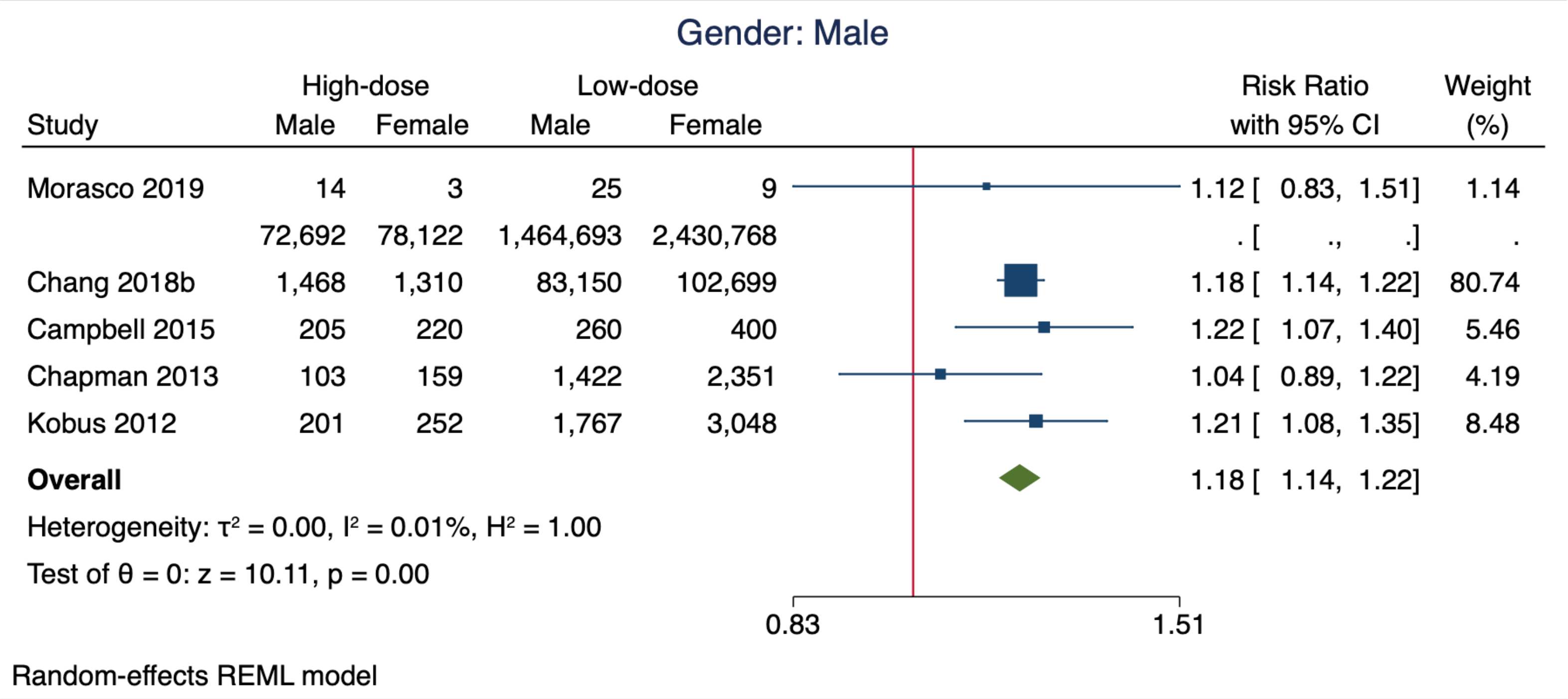

Figure S3. Forest plot of factors not associated with the prescribing of high-dose opioids in primary care

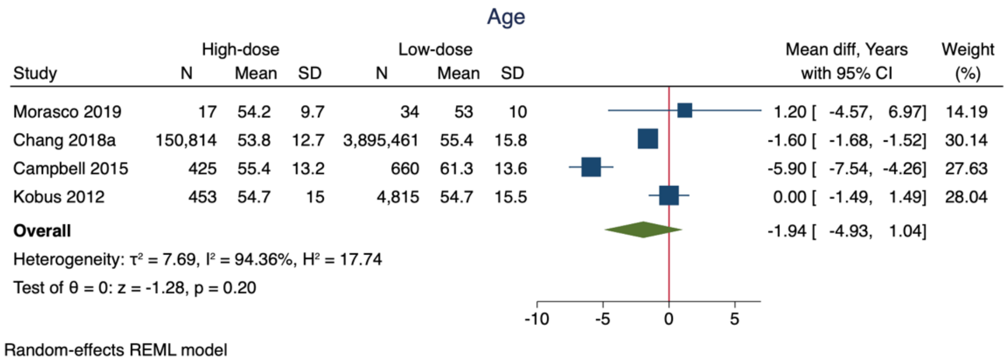

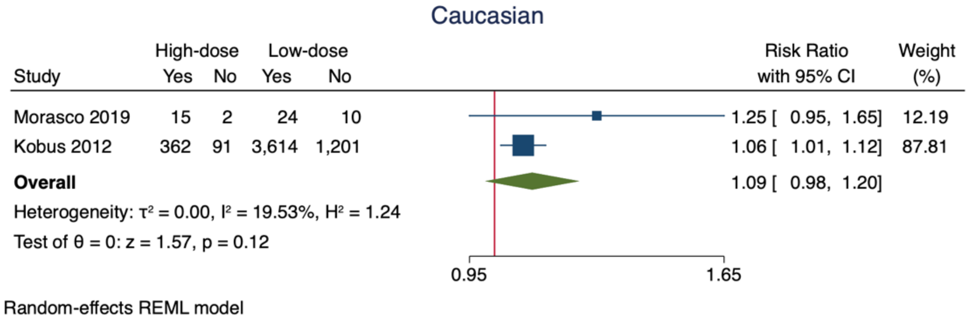

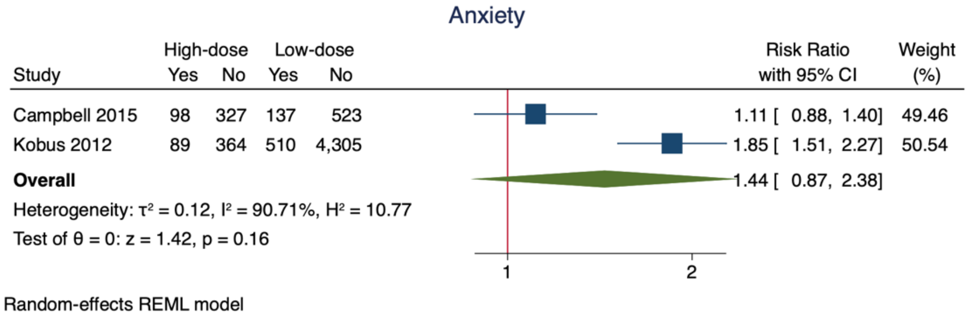

Table S5. Factors not associated with the prescribing of high-dose opioids reported by individual studies.

Study ID	Variable	High-dose	Low-dose	RR (95% CI)
		Count (%)	Count (%)	
**Sociodemographic characteristics**				
**Kobus, 2012**	Ethnicity<br>Black<br>Native American/ Alaskan<br>Asian Pacific Islander<br>Hispanic<br>Other	 7 (2%)<br>9 (2%)<br>4 (1%)<br>5 (2%)<br>7 (2%)	 134 (3%)<br>49 (1%)<br>40 (1%)<br>93 (3%)<br>99 (2%)	 0.56 (0.26, 1.18)<br>1.95 (0.97, 3.95)<br>1.06 (0.38, 2.96)<br>0.57 (0.23, 1.40)<br>0.75 (0.35, 1.61)
**Morasco, 2019**	Employment status<br>Employed<br>Receiving disability<br>Other	 1 (6%)<br>12 (71%)<br>1 (6%)	 3 (9%)<br>20 (60%)<br>4 (12%)	 0.67 (0.07, 5.94)<br>1.20 (0.79, 1.82)<br>0.50 (0.06, 4.13)
	Marriage status<br>Married<br>Separated/divorced<br>Single<br>Widowed	 7 (41%)<br>8 (41%)<br>2 (12%)<br>0 (0%)	 11 (32%)<br>16 (47%)<br>5 (15%)<br>2 (6%)	 1.27 (0.60, 2.69)<br>1.00 (0.54, 1.85)<br>0.80 (0.17, 3.71)<br>0.39 (0.02, 7.68)
**Kobus, 2012**	Insurance coverage<br>Medicaid	 1 (0.2%)	 3 (0.06%)	 3.54 (0.37, 33.99)
**Treatment-related characteristics**				
**Campbell, 2015**	Australian Schedule 4 drug	146 (34%)	197 (30%)	1.15 (0.97, 1.37)
	Over-the-counter analgesic use	259 (61%)	424 (64%)	0.95 (0.86, 1.04)
	Past week oxycodone	251 (59%)	400 (61%)	0.91 (0.82, 1.01)
	Past week buprenorphine	36 (8%)	209 (32%)	0.25 (0.18, 0.35)
	Past 3-month doctor shopping	6 (1%)	5 (1%)	1.86 (0.57, 6.07)
	Used other person's opioid medication past 3-months	12 (3%)	12 (2%)	1.55 (0.70, 3.42)
**Substance use**				
**Campbell, 2015**	Lifetime overdose on any substance	79 (19%)	103 (16%)	1.19 (0.91, 1.55)
	Past 12 months risky drinking (>5 standard drinks)	96 (23%)	165 (25%)	0.90 (0.73, 1.13)
**Physical health and patient behaviours**				
		**Median (IQR)**	**Median (IQR)**	**Mean difference**

**Kobus, 2012**	Comorbidity (RxRisk) score	895.9 (653–2,115)	895.9 (653–1,432)	0
		**Count (%)**	**Count (%)**	**RR (95% CI)**
	Smoking	256 (57%)	2,489 (52%)	1.09 (1.00, 1.19)
	BMI ≥ 30	235 (52%)	2,388 (50%)	1.05 (0.95, 1.15
**Clinical factors**				
**Campbell, 2015**	Type of pain<br>Arthritis or rheumatism<br>Visceral pain	 256 (60%)<br>96 (23%)	 424 (64%)<br>141 (21%)	 0.94 (0.85, 1.03)<br>1.06 (0.84, 1.33)
**Healthcare utilisation**				
**Kobus, 2012**	ED visit with back pain diagnosis	131 (29%)	1,348 (28%)	1.03 (0.89, 1.20)

Table S6. All factors reported by included studies

1. Sociodemographic characteristics

**1.1 Age**					
**Study ID**	**High-dose**		**Low-dose**		**Mean difference, years (95% CI)**
	**Mean age (years)**	**SD**	**Mean age (years)**	**SD**	
**Morasco, 2019**	54.2	9.7	53.0	10.0	1.20 (-4.6, 6.9)
**Chang, 2018a**	53.8	12.7	55.4	15.8	-1.6 (-1.7, -1.5)
**Chang, 2018b**	47.22	10.6	-	-	-
**Campbell, 2015**	55.4	13.2	61.3	13.6	-5.9 (-7.5, -4.3)
**Kobus, 2012**	54.7	15.0	54.7	15.5	0 (-1.5, 1.5)
**Chapman, 2013**	Age was reported for all participants on opioids, split by women and men. Women had a mean age of 67 years (SD: 17, range: 24 to 104 years) and men were 62 years (SD: 16, range: 19 to 97 years).				

**1.2 Gender (male)**			
	**High-dose**	**Low-dose**	**RR (95% CI)**
	**Count (%)**	**Count (%)**	
**Morasco, 2019**	14 (82%)	25 (74%)	1.12 (0.83, 1.51)
**Chang, 2018a**	72,692 (48%)	1,464,693 (38%)	1.28 (1.28, 1.29)
**Chang, 2018b**	1,468 (53%)	83,150 (44%)	1.18 (1.14, 1.22)
**Campbell, 2015**	205 (48%)	260 (39%)	1.22 (1.07, 1.40)
**Chapman, 2013**	103 (39%)	1,422 (38%)	1.04 (0.89, 1.22)
**Kobus, 2012**	201 (44%)	1,767 (37%)	1.21 (1.08, 1.35)

**1.3 Ethnicity**				
	**Variable**	**High-dose**	**Low-dose**	**RR (95% CI)**
		**Count (%)**	**Count (%)**	
**Morasco, 2019**	Caucasian	15 (88%)	24 (71%)	1.25 (0.95, 1.65)
**Kobus, 2012**	Caucasian	362 (80%)	3,614 (75%)	1.06 (1.01, 1.12)
**Kobus, 2012**	Black	7 (2%)	134 (3%)	0.56 (0.26, 1.18)
	Native American/ Alaskan	9 (2%)	49 (1%)	1.95 (0.97, 3.95)

	Asian Pacific Islander	4 (1%)	40 (1%)	1.06 (0.38, 2.96)
	Other	7 (2%)	99 (2%)	0.75 (0.35, 1.61)
	Unknown/declined to answer	64 (14%)	879 (18%)	0.77 (0.61, 0.98)
	Hispanic	5 (2%)	93 (3%)	0.57 (0.23, 1.40)
**1.4 Employment status**				
**Morasco, 2019**	Unemployed	3 (18%)	7 (21%)	0.86 (0.25, 2.91)
**Campbell, 2015**	Unemployed	245 (58%)	263 (40%)	1.45 (1.28, 1.64)
**Morasco, 2019**	Employed	1 (6%)	3 (9%)	0.67 (0.07, 5.94)
	Other	1 (6%)	4 (12%)	0.50 (0.06, 4.13)
	Receiving disability	12 (71%)	20 (60%)	1.20 (0.79, 1.82)
**1.5 Education**				
		**High-dose**	**Low-dose**	**Mean difference, years**
		**Mean (SD)**	**Mean (SD)**	
**Morasco, 2019**	Years of education	15.2 (2.6)	13.5 (2.0)	1.7
**1.6 Marriage status**				
**Morasco, 2019**	Married	7 (41%)	11 (32%)	1.27 (0.60, 2.69)
	Separated/ divorced	8 (41%)	16 (47%)	1.00 (0.54, 1.85)
	Single	2 (12%)	5 (15%)	0.80 (0.17, 3.71)
	Widowed	0 (0%)	2 (6%)	0.39 (0.02, 7.68)
**1.7 State of residence**				
**Chang, 2018a**	California<br>Florida<br>Georgia	47,446 (31%)<br>54,338 (36%)<br>20,692 (14%)	1,416,000 (36%)<br>1,207,982 (31%)<br>689,886 (18%)	0.87 (0.86, 0.87)<br>1.16 (1.15, 1.17)<br>0.77 (0.76, 0.78)

	Maryland	12,487 (8%)	250,868 (6%)	1.29 (1.26, 1.31)
	Washington	15,866 (11%)	330,335 (8%)	1.24 (1.22, 1.26)
**1.8 Insurance coverage**				
**Kobus, 2012**	Medicare	154 (34%)	1,352 (28%)	1.21 (1.06, 1.39)
	Medicaid	1 (0.2%)	3 (0.06%)	3.54 (0.37, 33.99)

2. Treatment-related characteristics

**2.1 Coprescription**				
**Study ID**	**Variable**	**High-dose**	**Low-dose**	**RR (95% CI)**
		**Count (%)**	**Count (%)**	
**Chang, 2018a**	Benzodiazepines	61,623 (41%)	314,364 (8%)	5.06 (5.03, 5.10)
**Chang, 2018b**	Benzodiazepines	1,226 (44%)	8,488 (5%)	9.36 (9.96, 10.28)
**Campbell, 2015**	Benzodiazepines	172 (40%)	169 (26%)	1.58 (1.33, 1.88)
**Kobus, 2012**	Sedative-hypnotic prescription 6-months before/ after index visit	276 (61%)	2,022 (42%)	1.45 (1.34, 1.57)
**Campbell, 2015**	Antidepressants	246 (58%)	323 (49%)	1.18 (1.06, 1.32)
**2.2 Opioid schedule**				
**Campbell, 2015**	Australian Schedule 4	146 (34%)	197 (30%)	1.15 (0.97, 1.37)
	OTC analgesic use	259 (61%)	424 (64%)	0.95 (0.86, 1.04)
**Kobus, 2012**	Long-acting	400 (88%)	1,637 (34%)	2.60 (2.47, 2.74)
**2.3 Type of opioid drug**				
**Campbell, 2015**	Past week oxycodone	251 (59%)	400 (61%)	0.91 (0.82, 1.01)
	Past week morphine	86 (20%)	75 (11%)	1.78 (1.34, 2.37)
	Past week buprenorphine	36 (8%)	209 (32%)	0.25 (0.18, 0.35)
**2.4 Adverse events and adverse drug reactions**				
		**High-dose**	**Low-dose**	**Statistical analysis**
		**Median (IQR)**	**Median (IQR)**	
**Campbell, 2015**	Number of adverse	91-199: 5	21-90: 4 (1-	Not possible

	events	(2-9.5)<br>≥200: 6 (3-11)	7)<br>1-20: 2 (0-6)	
		**Count (%)**	**Count (%)**	**RR (95% CI)**
**Campbell, 2015**	ICD-10 lifetime pharmaceutical opioid dependence	49 (12%)	28 (4%)	2.72 (1.7, 4.25)
	ICD-10 12-month pharmaceutical opioid dependence	26 (6%)	13 (2%)	3.11 (1.61, 5.98)
**2.5 Duration of opioid use**				
		**High-dose**	**Low-dose**	**Statistical analysis**
		**Median (IQR)**	**Median (IQR)**	
**Campbell, 2015**	Years prescribed opioids	91-199: 6 (2-13)<br>≥200: 7.8 (3-15)	21-90: 3 (1.1-8)<br>1-20: 2.5 (0.6-5)	Not possible
**2.6 Opioid treatment problems and risks**				
		**High-dose**	**Low-dose**	**Mean difference**
		**Mean (SD)**	**Mean (SD)**	
**Morasco, 2019**	Risk for prescription opioid misuse from the Pain Medication Questionnaire	26.6 (6.4)	25.3 (9.2)	1.3
		**Count (%)**	**Count (%)**	**RR (95% CI)**
**Campbell, 2015**	Prescribed opioid difficulty scale (PODS) intermediate- high (≥8)	297 (70%)	367 (56%)	1.26 (1.15, 1.38)
**2.7 Treatment-related behaviours**				
		**High-dose**	**Low-dose**	**RR (95% CI)**
		**Count (%)**	**Count (%)**	
**Campbell, 2015**	Past 3-month tampering	38 (9%)	29 (4%)	2.03 (1.27, 3.25)
	Past 3-month doctor shopping	6 (1%)	5 (1%)	1.86 (0.57, 6.07)
	Past 3-month different drug route	7 (2%)	1 (0.2%)	10.87 (1.34, 88.04)
	Used other person's	12 (3%)	12 (2%)	1.55 (0.70, 3.42)

	opioid medication past 3-months			
**2.8 Number of opioid drugs**				
		**High-dose**	**Low-dose**	**Statistical analysis**
		**Median (IQR)**	**Median (IQR)**	
**Campbell, 2015**	Number of opioid drugs	91-199: 1 (1-1)<br>≥200: 1 (1-2)	21-90: 1 (1-1)<br>1-20: 1 (1-1)	Not possible

Clinical Characteristics
3. Substance use

**3.1 Illicit drug use**				
**Study ID**	**Variable**	**High-dose**	**Low-dose**	**RR (95% CI)**
		**Count (%)**	**Count (%)**	
**Campbell, 2015**	Illicit drug use past 12 months	71 (17%)	67 (10%)	11.03 (5.75, 21.14)
**3.2 Non-illicit substance use**				
		**Mean (SD)**	**Mean (SD)**	**Mean difference**
**Morasco, 2019**	Days of alcohol use in past 30 days	0.6 (1.3)	2.1 (5.1)	-1.5
**3.3 Substance use problems/disorders**				
		**Count (%)**	**Count (%)**	**RR (95% CI)**
**Chang, 2018b**	Opioid disorders	530 (19%)	1,243 (1%)	28.95 (26.34, 31.82)
**Campbell, 2015**	Lifetime overdose on any substance	79 (19%)	103 (16%)	1.19 (0.91, 1.55)
**Kobus, 2012**	Substance use disorder	141 (31%)	1,151 (24%)	1.30 (1.13, 1.51)
		Mean (days)	Mean (days)	
**Chang, 2018b**	Magnitude of high-risk use in 2012	115.7	-	-
**3.4 Potential substance use problems**				
		**Count (%)**	**Count (%)**	**RR (95% CI)**
**Campbell, 2015**	Past 12 months risky drinking (>5 standard drinks)	96 (23%)	165 (25%)	0.90 (0.73, 1.13)

4. Physical health and patient behaviours

4.1 Morbidity measures						
**Study ID**	**Variable**	**High-dose**		**Low-dose**		**Mean difference**
		**Mean score**	**SD**	**Mean score**	**SD**	
**Chang, 2018a**	Chronic disease score	27.3	21.8	13.3	13.9	14
**Chang, 2018b**	Aggregated diagnostic cluster morbidity group (ADG) 2012	8.4	4.3	-	-	-
	Aggregated diagnostic cluster morbidity group (ADG) 2013	8.3	4.5	-	-	-
	Rx-defined morbidity groups (Rx-MGs) 2012	8.9	4.5	-	-	-
	Rx-defined morbidity groups (Rx-MGs) 2013	8.8	10.0	-	-	-
	Count of chronic conditions 2012	4.0	3.1	-	-	-
	Count of chronic conditions 2013	4.0	3.3	-	-	-
	Active ingredients 2012	13.1	8.2	-	-	-
	Active ingredients 2013	12.8	8.3	-	-	-
	Concurrent risk score 2012	4.7	6.8	-	-	-
	Concurrent	4.8	47.4	-	-	-

	risk score 2013					
		**Median**	**IQR**	**Median**	**IQR**	**Mean difference**
**Kobus, 2012**	Comorbidity (RxRisk) score	895.9	653–2,115	895.9	653–1,432	0
**4.2 Smoking**						
**Study ID**		**High-dose**		**Low-dose**		**RR (95% CI)**
		**Count (%)**		**Count (%)**		
**Morasco, 2019**		30.7% of the sample endorsed smoking cigarettes				Not possible
**Kobus, 2012**		256 (57%)		2,489 (52%)		1.09 (1.00, 1.19)
**4.3 Body Mass Index (BMI)**						
**Kobus, 2012**	BMI ≥ 30	235 (52%)		2,388 (50%)		1.05 (0.95, 1.15
**4.4 Physical health score**						
		**High-dose**		**Low-dose**		**Statistical analysis**
		**Median (IQR)**		**Median (IQR)**		
**Campbell, 2015**	SF-12	91-199: 25.9<br>≥200: 25.8		21-90: 26.6<br>1-20: 28.1		Not possible

5. Pain measures

**5.1 Duration of pain**				
**Study ID**	**Variable**	**High-dose**	**Low-dose**	**RR (95% CI)**
		**Median (IQR)**	**Median (IQR)**	
**Campbell, 2015**	Years living with pain	91-199: 11 (5-22)<br>≥200: 15 (5-12)	21-90: 10 (2-21)<br>1-20: 11 (3-23)	Not possible
	12 month chronic pain conditions	91-199: 2 (2-3)<br>≥200: 2 (2-3)	21-90: 2 (1-3)<br>1-20: 2 (1-3)	Not possible
**5.2 Pain measures**				
		**High-dose**	**Low-dose**	**Linear regression model**
		**Slope of function, k**	**Slope of function, k**	
**Morasco, 2019**	Delay discounting	-5.8 (2.3)	-4.8 (2.0)	DD was significantly associated with

				dose (p-value: 0.003)
		**Mean (SD)**	**Mean (SD)**	**Mean difference**
**Morasco, 2019**	Pain severity from the Multi-dimensional Pain Inventory	4.5 (1.0)	4.1 (0.9)	0.40
	Pain interference from the Multi-dimensional Pain Inventory	4.7 (1.0)	4.6 (1.0)	0.10
		**Mean (SD)**	**Mean (SD)**	**RR (95% CI)**
**Campbell, 2015**	Pain severity from the Brief Pain Inventory	91-199: 5.4 (1.6)<br>≥200: 5.4 (1.8)	21-90: 4.8 (1.8)<br>1-20: 4.4 (1.8)	1.21 (1.11, 1.31)<br>1.21 (1.1, 1.35)
	Pain interference score from the Brief Pain Inventory	91-199: 6.1 (2.1)<br>≥200: 6.2 (2)	21-90: 5.3 (2.3)<br>1-20: 4.7 (2.3)	1.18 (1.09, 1.26)<br>1.23 (1.13, 1.34)
	Pain self-efficacy (PSEQ)	91-199: 27.4 (12.7)<br>≥200: 24.7 (12.5)	21-90: 31.8 (12.)<br>1-20: 35.8 (13.8)	0.97 (0.96, 0.99)<br>0.96 (0.94, 0.97)
**5.3 Type of pain conditions**				
		**High-dose**	**Low-dose**	**RR (95% CI)**
		**Count, %**	**Count, %**	
**Campbell, 2015**	Arthritis or rheumatism	256 (60%)	424 (64%)	0.94 (0.85, 1.03)
	Back or neck problems	344 (81%)	484 (73%)	1.10 (1.03, 1.18)
	Frequent/severe headaches	134 (32%)	170 (26%)	1.22 (1.01, 1.48)
	Visceral pain	96 (23%)	141 (21%)	1.06 (0.84, 1.33)

6. Healthcare utilisation

**6.1 Clinic visits**

Study ID	Variable	High-dose	Low-dose	Median difference
		Median	Median	
**Kobus, 2012**	Clinic visits of any type 6 months before/after index date	22	17	5
		**Count (%)**	**Count (%)**	**RR (95% CI)**
**Kobus, 2012**	Any pain clinic visit 6 months before/after index date	104 (23%)	530 (11%)	2.09 (1.73, 2.51)
**6.2 Secondary & tertiary care use**				
		**High-dose**	**Low-dose**	**RR (95%)**
		**Count (%)**	**Count (%)**	
**Chang, 2018b**	>1 emergency visit in 2012	829 (30%)	51,534 (27%)	1.09 (1.03, 1.16)
	>1 emergency visit in 2013	765 (28%)	34,338 (18%)	1.51 (1.42, 1.61)
**Kobus, 2012**	ER visit 6 months before/ after index visit	277 (50%)	1,878 (39%)	1.57 (1.45, 1.70)
**Chang, 2018b**	>1 hospitalisation in 2012	443 (16%)	17,061 (9%)	1.76 (1.62, 1.92)
	>1 hospitalisation in 2013	396 (14%)	11,110 (6%)	2.42 (2.21, 2.66)
		**Mean (SD)**	**Mean (SD)**	**Mean difference**
**Kobus, 2012**	Hospitalisation 6 months before/ after index date	1.9 (1.3)	1.5 (1.1)	0.4
		**Count (%)**	**Count (%)**	**RR (95%)**
	ED visit with back pain diagnosis	131 (29%)	1,348 (28%)	1.03 (0.89, 1.20)
	Filled opioid prescription 5 days after ED visit	285 (63%)	2,696 (56%)	1.12 (1.04, 1.21)

6.3 Multiple prescribers				
**Chang, 2018a**	Obtain opioids from ≥ 4 unique prescribers & pharmacies over a 90 day period	1,176 (0.78%)	1,948 (0.05%)	15.6 (14.51, 16.76)
		**Median**	**Median**	**Median difference**
**Kobus, 2012**	Median opioid prescribers	4	3	1 (p=<0.001)
**6.4 Healthcare costs**				
		**$ (USD)**	**$ (USD)**	
**Chang, 2018b**	Total concurrent cost (2012)	30,486	-	-
	Total prospective cost (2013)	31,045	-	-
	Medical cost 2012	19,275	-	-
	Medical cost 2013	19,663	-	-
	Pharmacy cost 2012	11,211	-	-
	Pharmacy cost 2013	11,382	-	-
	Opioid medication cost 2012	6,169	-	-
	Opioid medication cost 2012	6,079	-	-

7. Mental health

7.1 Depression				
		**High-dose**	**Low-dose**	

Study ID	Variable & measure/metric	Count (%)	Count (%)	RR (95%)
**Campbell, 2015**	Moderate-severe depression [score of 10 on the PHQ-9]	221 (52%)	256 (39%)	1.34 (1.17, 1.53)
**Kobus, 2012**	ICD-9 diagnostic codes for depression: 296.2, 296.3, 300.4, 309.0, 309.1, 311	190 (42%)	1,425 (30%)	1.42 (1.26, 1.59)
		**Mean (SD)**	**Mean (SD)**	**Mean difference**
**Morasco, 2019**	Severity of depressive symptoms using the Beck Depression Inventory-2	21.5 (12.1)	20.0 (13.7)	1.5
**7.2 Anxiety**				
		**High-dose**	**Low-dose**	**Mean difference**
		**Mean (SD)**	**Mean (SD)**	
**Morasco, 2019**	Severity of anxiety symptoms using the Generalized Anxiety Disorder-7	9.9 (5.6)	9.9 (6.0)	0
		**Count (%)**	**Count (%)**	**RR (95%)**
**Campbell, 2015**	Moderate-severe anxiety	98 (23%)	137 (21%)	1.14 (0.85, 1.53)
**Kobus, 2012**	ICD-9 diagnostic codes for anxiety: 300.0 – 300.09	89 (20%)	510 (11%)	2.06 (1.61, 2.65)
**7.3 Post-traumatic stress disorder (PTSD)**				
		**High-dose**	**Low-dose**	
		**Count (%)**	**Count (%)**	**RR (95%)**
**Kobus, 2012**	Posttraumatic stress disorder diagnostic code 309.81	20 (4%)	96 (2%)	2.21 (1.38, 3.55)

7.4 Any of depression, anxiety, PTSD or substance use				
**Kobus, 2012**	Report of depression, anxiety, PTSD and/or substance use disorder	280 (62%)	2,263 (47%)	1.32 (1.22, 1.42)

8. Patient beliefs

8.1 Relief from current medicines			
**Study ID**	**High-dose**	**Low-dose**	**Statistical analysis**
	**Median (IQR)**	**Median (IQR)**	
**Campbell, 2015**	91-199: 6 (5-8)<br>≥200: 6 (5-8)	21-90: 7 (5-8)<br>1-20: 7 (5-8)	Not possible

9. Prescriber behaviours

9.1 High-risk prescribers						
**Study ID**	**Variable**	**High-dose**		**Low-dose**		**RR (95% CI)**
		**Mean %**	**SD**	**Mean %**	**SD**	
**Chang, 2018a**	Proportion of prescriptions from high-risk prescribers	122,159 (81%)	31.8	973,865 (25%)	39.4	3.24 (3.23, 3.25)
**Percentile group**		**Count (%)**		**Count (%)**		**RR (95% CI)**
**Chang, 2018a**	100% (all opioid prescriptions from high-risk prescribers)	77,217 (51%)		572,633 (15%)		3.48 (3.46, 3.50)
	50-99% of prescriptions from high-risk prescribers	51,277 (34%)		471,351 (12%)		2.81 (2.79, 2.83)
	1-49% of prescriptions from high-risk prescribers	8,747 (6%)		222,041 (6%)		0.10 (0.10, 0.10)
	0% (no prescriptions from high-risk prescribers)	13,573 (9%)		2,629,436 (68%)		0.13 (0.13, 0.15)
**9.2 High-volume prescribers**						
**Study ID**	**Variable**	**Mean**	**SD**	**Mean**	**SD**	**Mean difference**
**Chang, 2018a**	Daily opioid dose per transaction	120	70.8	48	42.7	72
	Days supplied per transaction	27	6.8	25	8.3	2
	Opioid volume per person	56	36.4	3	7.0	53

	Opioid prescription per person	18	11.0	2	5.0	16
		**Count (%)**		**Count (%)**		**RR (95% CI)**
**Chang, 2018a**	Proportion of total opioid volume	50,975 (34%)		1,488,066 (38%)		0.88 (0.88, 0.89)
	Proportion of total opioid prescriptions	16,590 (11%)		1,406,261 (36%)		0.31 (0.30, 0.31)
**9.3 Low-volume prescribers**						
		**High-dose**		**Low-dose**		**Mean difference**
		**Mean**	**SD**	**Mean**	**SD**	
**Chang, 2018a**	Daily opioid dose per transaction	102	70.2	35	27.9	67
	Days supplied per transaction	23	10.2	15	11.2	8
	Opioid volume per person	10	21.4	1	3.6	9
	Opioid prescription per person	4	7.5	3	4.0	1
		**Count (%)**		**Count (%)**		**RR (95% CI)**
**Chang, 2018a**	Proportion of total opioid volume	8,747 (6%)		864,792 (22%)		0.26 (0.26, 0.27)
	Proportion of total opioid prescriptions	3,620 (2%)		1,967,208 (51%)		0.05 (0.05, 0.05)
